# Education of complementary and alternative medicine in adult education centers in Germany: a web-based survey

**DOI:** 10.1007/s10354-022-00951-0

**Published:** 2022-08-10

**Authors:** Kai-Uwe Ott, Christian Keinki, Lukas Kaesmann, Jutta Huebner

**Affiliations:** 1 Department of Cardiology, Kerckhoff-Klinik, Benekestr. 2–8, 61231 Bad Nauheim, Germany; 2 Practice for general medicine Dres.Ott&Schoening, Karlstr. 3, 63579 Freigericht, Germany; 3 Department of Hematology and Oncology, Jena University Hospital, Am Klinikum 1, 07747 Jena, Germany; 4 Department of Radiation Oncology, University Hospital, LMU Munich, Marchioninistr. 15, 81377 Munich, Germany; 5 German Cancer Consortium (DKTK), partner site Munich, Munich, Germany

**Keywords:** Health literacy, Nutrition, Diet, Adult education, Non-medical practitioners

## Abstract

**Background:**

Adult education centers are an important part of health education worldwide. Our aim was to evaluate the courses offered by German adult education centers with regard to complementary medicine and nutrition.

**Methods:**

A systematic web-based search was done for the websites of German adult education centers and courses were analyzed considering topics, scientific soundness, and qualification of instructors.

**Results:**

Our search revealed 502 courses, 360 (71.7%) related to complementary and alternative medicine (CAM) and 176 courses on nutrition (35.1%). CAM courses most often presented methods with a focus on traditional Eastern medicine with yoga and similar mind–body practices (41.9%), traditional Chinese medicine (TCM; 13.3%), and ayurvedic medicine (11.4%). Content concerning nutrition mainly included controversial fasting methods like alkaline fasting, detox diets, and therapeutic fasting (43.8%), as well as Eastern traditional diets from TCM and ayurveda (21.7%). Most of the courses were given by non-medical practitioners (NMPs; 36.4%), while only very few physicians were engaged.

**Conclusion:**

There are substantial doubts on the scientific background of many courses offered for lay adult health education. Besides direct misinformation, many courses reinforce alternative and non-evidence-based notions in society. Adult education centers should reconsider the topics of their courses as well as the professional qualifications of the instructors.

## Introduction

Adult education centers are working as educational institutions on a community level in Germany. The courses they offer play an important role in civic education on different topics and give their participants the opportunity of lifelong learning.

Courses on health education are the most popular in Germany, with around 2.5 million participants per year [1].

Complementary and alternative medicine (CAM) has become an essential addition to conventional medical treatments for many patients. In Germany, the percentage of people having experience with CAM methods is 26–73% [2]; across Europe, every second person has been in touch with CAM methods [3], whereas 95% have used at least one CAM treatment in the US [4]. Although the range of CAM methods shows a high diversity, some of the methods are recognized by conventional medicine and become integrated into medical guidelines.

Patients’ and citizens’ knowledge of medical issues and their health literacy has an important impact on individual as well as collective decisions in health care [5].

Accordingly, our aim is to evaluate the content of educational offers of adult education centers in Germany, which focus on topics which patients typically associate with CAM.

Because of their important role in medical education and many content-related overlaps with CAM, especially in plant-based foods, food supplements, and medical plants, we decided to integrate courses with relation to nutrition and dieting as well.

As far as we know, this survey is the first of its kind and gives a representative overview on the course offers of German adult education centers concerning CAM and nutrition; moreover, it considers their didactical concepts for potential improvement.

## Methods

For our analysis, we reviewed courses offered by German adult education centers with content related to CAM or nutrition in a period between September 2020 and September 2021. For selection of the adult education centers, we chose Germany’s ten largest cities by population and set a search radius of 80 km. Using an internet-based search via Google, we used the general terms complementary and alternative medicine, and a more detailed vocabulary with the most used or offered CAM methods in Germany [6].

Similarly, in addition to the courses related to CAM, we proceeded with courses on nutrition. Within our web-based search, we used the general terms nutrition and fasting as well as more detailed terms. An alphabetical order of our search terms is shown in Table 1.

**Table 1:** Overview of the search terms used in alphabetical order

*Complementary and alternative medicine*	Acupressure
	Acupuncture
	Alternative medical/alternative medicine
	Anthroposophiological/antroposophy
	Aromatherapy
	Bach flowers
	Bioresonance
	Blood letting
	Complementary medical/complementary medicine
	Constellations
	Cupping
	Dark field diagnostics
	Enderlein diagnostics
	Gem therapy
	High tone therapy
	Leech therapy
	Own blood
	Urine therapy
	Reflexology
	Iridology
	Iris diagnosis
	Kinesiology
	Light therapy
	Luescher diagnostics
	Mitochondrial therapy
	Naturopathical/naturopathy
	Ozone therapy
	Oxygen therapy
	Pendulum therapy
	Schuessler salts
	Spagyric
	Traditional Chinese Medicine
*Nutrition*	Acid-base-balance
	Detoxifying
	Draining therapy
	Intestinal sanation
	Fasting
	Low-carbohydrate nutrition
	Orthomolecular medicine
	Orthomolecular treatment
	Nutritional therapy
	Symbiosis
	Vitamins

Data were recorded in a Microsoft Excel (Microsoft, Redmond, WA, USA) table, including title of the course, city (place and zip code), name and professional position of the course instructor, and link to the adult education center’s website. In a second step, we removed courses which included health topics without naming a specific CAM method or specific information on nutrition.

## Further analysis

### Course instructors

Based on the information shared on the website of each adult education center, we documented the course instructor’s professional function and assessed information on their expertise on CAM or nutrition.

### Complementary and alternative medicine

The courses with respect to CAM were sorted into five overarching topic areas with 11 different categories. (Table 2).

**Table 2:** Course offers of adult education centers on complementary and alternative medicine grouped into topic areas

Topic area	Category
Body and musculoskeletal system	Massage therapy/fascia treatment
	Reflexology
	Mind and body practices
Psyche, spirit, and social interaction	Relaxation techniques
	Energy field methods
	Constellation therapy
	Mind and body practices
Traditional Far Eastern medicine	Traditional Chinese medicine
	Ayurvedic medicine
Naturopathy	General naturopathic treatment
	Phytotherapy/herbs/tea mixtures
Homeopathy	Homeopathy

These categories are not exclusive, for example, the course *Anti-Aging mit Yoga und Ayurveda* (Anti-Aging with Yoga and Ayurveda) is assigned to both the mind–body procedure and the ayurveda category. The assignment of each individual procedure to a category is shown in Table 3.

**Table 3:** Courses of adult education centers on complementary and alternative medicine matched to categories

Category	Course offers
Mind and body practices	Yoga, tai-chi, qigong, xing yi quan, meditation
Reflexology	Standard reflexology, hand reflexology, foot reflexology
Massage therapy/fascia treatment	Classic massage treatment, Chinese and Japanese massage techniques, neurological fascia treatment, Dorn method
Relaxation techniques	Autogenic training, progressive muscle relaxation, mindfulness-based stress reduction, imaginative therapy
Energy field methods	Bioenergetics, bioenergetic analysis, chakra methods, kinesiological treatment
Constellation therapy	Systemic constellations, family constellations
General naturopathic treatment	General naturopathic methods for internal, gynecological, urologic, and orthopedic disease pattern, Hildegard of Bingen’s medicine, compresses and fomentation, forest bathing
Phytotherapy/herbs/tea mixtures	Phytotherapy, herbs and ointments, tea mixtures
Traditional Chinese medicine	Acupuncture/acupressure, pharmacotherapy in TCM, massage therapy in TCM, traditional Chinese diets
Ayurvedic medicine	Ayurvedic massage techniques, traditional ayurvedic diet, ayurvedic phytopharmacy
Homeopathy	Classic homeopathy, complex homeopathy, Schuessler salts

*TCM* Traditional Chinese Medicine

### Nutrition

Courses were then matched to six categories (Table 4). As well as the course offers on CAM, offers on nutrition could be matched to several categories. Courses with relation to CAM and additional focus on nutrition were categorized in both groups.

**Table 4:** Courses of adult education centers on nutrition matched to categories

Category	Course topics
Nutrition in general	Nutritional theories, disease-related nutritional therapy, cooking recipes
Traditional diets	Traditional Chinese diet, Indian and ayurvedic diet
Intestine and microbiome	Diet and microbiome, nutrition, and functional intestinal complaints
Micronutrients	Vitamins, minerals, secondary plant substances
Low-carb diets	Low-carb diets
Fasting/detoxification/acid-base balance	Therapeutic fasting, intermittent fasting, detox diets, alkaline fasting

## Results

### Basic data of the courses

**Fig. 1:**
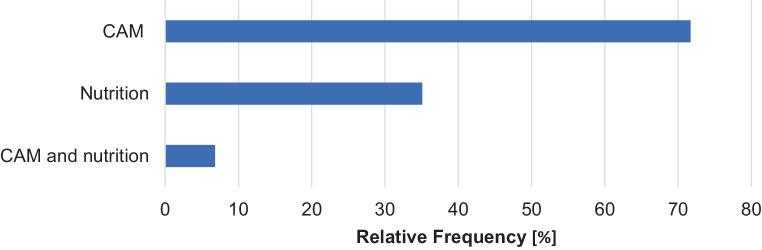
Distribution of the courses according to thematic focus. *CAM* complementary and alternative medicine

As part of the research, a total of 502 offers were considered which could be assigned to the fields of CAM or nutrition. With 71.7%, most of the offers could be assigned to one of these topics, 35.1% belonged to nutrition, and 6.8% could be subsumed in both groups. The distribution of the courses according to their thematic focus is shown in Fig. 1.

The geographical distribution of the courses shows many more offers in former West German federal states (76.9%) and only 23.1% in the former East German federal states (Table 5). At the time of our statistical analysis, 12.5 million people lived in the former East German federal states (Berlin not included), while around 70.66 million people lived in the former West German federal states (including Berlin) [7]. Thus, the density of offers is comparable in both parts of Germany.

**Table 5:** Geographical distribution of the course offers by federal state

Federal state	Number of courses	Percentage (%)
Saxony	19	3.8
Brandenburg	16	3.2
Thuringia	27	5.4
Saxony-Anhalt	9	1.8
Mecklenburg-Western Pomerania	6	1.2
*Former East German federal states (total)*	*77*	*15.3*
Berlin	39	7.8
Hamburg	12	2.4
Schleswig-Holstein	10	2.0
Lower Saxony	7	1.4
Bremen	0	0
North Rhine-Westphalia	156	31.1
Hesse	16	3.2
Rhineland-Palat	11	2.2
Bavaria	71	14.1
Saarland	0	0
Baden-Wuerttemberg	103	20.5
*Former West German federal states (total)*	*425*	*84.7*

### Course instructors

At the time of the study, 286 courses were listed with the name of a course instructor. In 72 courses (25.1%) no detailed information on the relevant professional qualifications could be obtained. For the remaining 214 courses, 63.1% of course instructors claimed to have knowledge in the fields of CAM or nutrition. While 36.4% work as non-medical practitioners (NMPs), physicians (2.8%), physiotherapists (2.3%), psychologists (0.9%), and psychotherapists (0.9%) were comparatively rare among the course instructors (Table 6). Professional qualifications without direct reference to CAM or nutrition were found in 9.3% of the cases.

**Table 6:** Professional qualifications of the course instructors

Professional qualification	Number	Percentage (%)
Knowledge of CAM or nutrition	135	63.1
Non-medical practitioner	78	36.4
Physician	6	2.8
Physiotherapist	5	2.3
Psychologist	2	0.9
No direct professional reference to CAM or nutrition	20	9.3

*CAM* Complementary and alternative medicine

The most often named expertise of the instructors in CAM was tai chi/qigong (31.9%), ayurveda (17.0%), yoga (15.6%), phytotherapy (14.8%), and reflexology and massage therapies (12.6%). Nutritional expertise was claimed by 25.2% (Table 7).

**Table 7:** Course instructors’ knowledge of complementary and alternative medicine or nutritional therapy

Subject area	Number	Percentage (%)
Tai chi/qigong	43	31.9
Nutritional advice	34	25.2
Ayurveda	23	17.0
Yoga	21	15.6
Phytotherapy	20	14.8
Reflexology and massage therapy	17	12.6
Relaxation techniques	13	9.6
Acupuncture/acupressure	10	7.4
Traditional Chinese medicine	9	6.7
Fitness	2	1.5
Other subject areas	9	6.7

### Complementary and alternative medicine

**Fig. 2:**
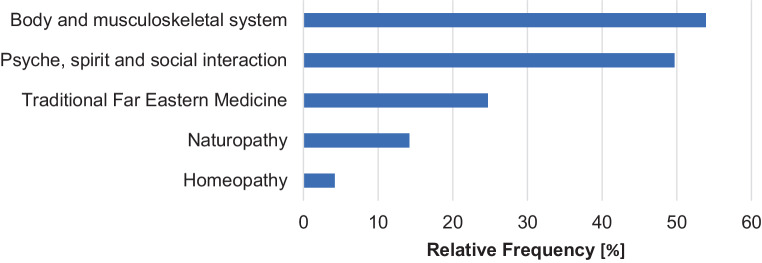
Overarching content-related topics for courses with reference to complementary and alternative medicine

In total, 360 courses (51.7%) could be assigned to the subject area of CAM. The majority of the courses were related to the topics “body and musculoskeletal system” (53.9%) and “psyche, spirit, and social interaction” (49.7%). A quarter (24.7%) belonged to the topic “traditional Far Eastern healing arts” (Fig. 2).

**Fig. 3:**
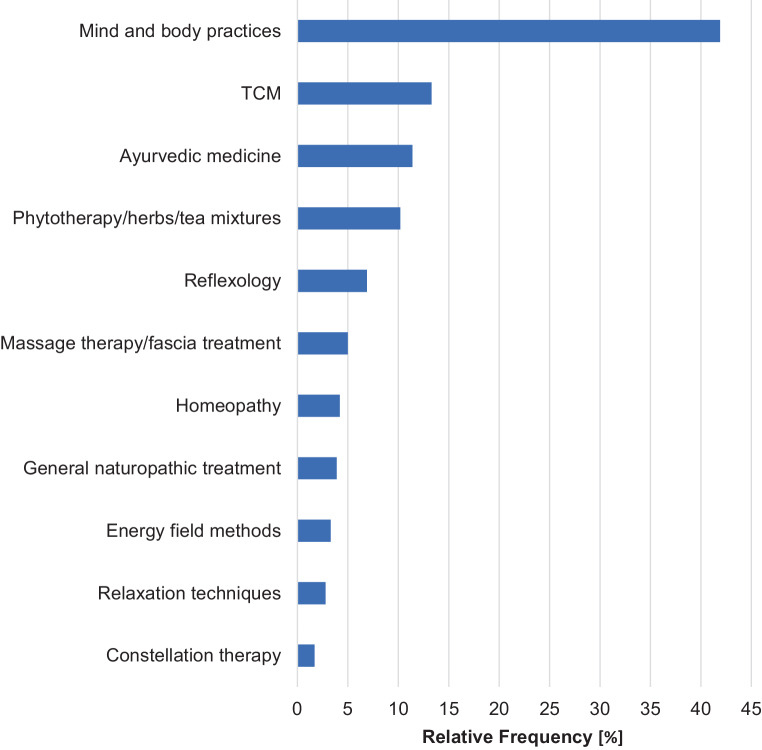
Categories to complementary and alternative medicine-related courses. *TCM* traditional Chinese medicine

With a frequency of 41.9%, mind–body procedures were the most common category among the courses with relation to CAM. Less often, traditional Chinese medicine (13.3%) and ayurveda (11.4%) as well as phytotherapy/herbs/tea mixtures (10.3%) were listed (Fig. 3).

### Nutrition

**Fig. 4:**
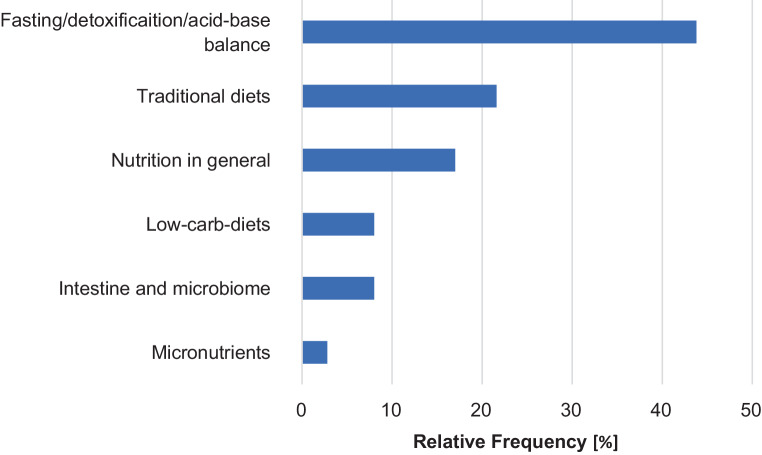
Nutrition courses according to categories

Nutrition was addressed in 276 courses (35.1%). The categories “fasting/detoxification/acid-base-balance” (43.8%) and “traditional forms of nutrition” (21.6%) were most often listed, followed by “general nutrition” (17.0%) (Fig. 4).

## Discussion

In summary, a wide range of topics are offered in adult education centers in Germany. We did not find substantial differences between offers in the western and eastern parts of Germany. This is remarkable, as in contrast to the development in the Federal Republic of Germany (FRG), medical services from non-medical practitioners as well as CAM were not wanted in the German Democratic Republic (GDR) [8]. Thus, until 1989, distribution of CAM was rare in the GDR. Within the past 30 years, this difference seems to have vanished in part. This rapid development points to a high demand for information on this specific content.

Courses offered by adult education centers in the category CAM are mostly based on topics of alternative medicine and Far Eastern healing arts. Instead, courses related to evidence-based complementary medicine and phytotherapy are rarely offered. In the category of nutrition, courses relating to the basics of healthy nutrition are seldom offered. In contrast, courses on special forms of diet prevail. A substantial portion of these courses represents controversial and non-evidence-based types of diet. Often, we found offers for alkaline fasting and traditional nutrition forms from traditional Chinese medicine or ayurveda.

While most of the courses were led by non-medical practitioners, courses led by healthcare professionals such as physicians, physiotherapists, or psychologists were rare. Frequently, special knowledge in CAM or nutrition was claimed by the course instructors. The lack of statues in training of NMPs and the diversity of offers on the market for additional specialist knowledge cast strong doubt regarding whether this is a qualifying further education. In fact, an analysis of the NMPs’ schools in has shown that the content taught by these schools is not scientific or evidence based [9].

Similar problems can also be found in the field of nutritional therapy. In Germany, several terms describing nutritional therapists, e.g., “nutritionist,” are not subject to any legal protection, which is why they can be used by everyone without further requirements. In addition to certified degrees, for example certified by the German Nutrition Society, there is also a variety of offers for private or institute-internal degrees [10]. Certified degrees in nutritional counseling or therapy could rarely be found in our study.

Highly problematic is that this lack of qualification might not be recognized by the participants of the courses who most probably rely on the selection criteria of the institutions. Due to the growing popularity, around 2.5 million participants per year enroll in health courses in adult education centers in Germany [1]. In fact, these courses receive a high level of acceptance by health insurance funds, mostly cost-covered and actively promoted by them [11].

Our analysis of course offers of German adult education centers with respect to CAM showed a thematic focus on mind and body practices, including mainly procedures from a Far Eastern cultural background such as tai chi, qigong, and yoga. Mind and body practices use the mutual influence of body, psyche, and behavior on health. The focus of this concept is to improve health resources and strengthen self-efficacy. Recent studies could describe positive effects for only a few indications such as coronary heart disease, arterial hypertension, and chronic lower back pain [12–14]. Despite a great public and media interest, an advantage of CAM over physical activity or sports and a relevant clinical impact have not been shown so far. Accordingly, it has been shown that the use of yoga interventions compared to sporting activity does not offer any advantages in terms of tumor-associated fatigue [15].

In fact, also for other methods offered, scientific evidence is missing, although individual health-promoting effects are sometimes described. This applies, for example, to the use of reflexology in cancer care or treatment of multiple sclerosis [16, 17]. The same is true for the use of massage therapy in supportive care for cancer patients [18] or reducing symptoms of fibromyalgia [19].

This also applies to most other TCM methods. While for acupuncture and acupressure many studies have been published, data are heterogenous [20–23]. In addition, relaxation techniques may reduce anxiety and depression of cancer patients and increase patients’ quality of life [24, 25].

As, most probably, the scientific evidence is scarcely discussed in the courses, non-evidence-based knowledge may strongly impact the medical understanding of larger parts of the population, which could encourage an improper use of CAM and diet methods.

Moreover, these methods gain impact as they impress patients by their ease of use and the allegedly missing side effects. Moreover, TCM is a good example of CAM methods not lacking side effects. Besides reports of hepato- and nephrotoxicity, some herbal or dietary supplements are mixed with pesticides or drugs like corticosteroids. These interactions with conventional therapy have to be co considered [26–28]. CAM methods should not be recommended for every user without considering the individual medical condition and circumstances. Potential side effects should also be discussed within the courses. Especially the uncritical use of medicinal plants and homeopathy can be associated with an increased health risk [29, 30].

The effectiveness of dieting is clinically well proven, especially in combination with exercise and behavioral therapy for overweight and obesity [31]. The aim of dieting methods is to improve or stabilize health.

Overall, course offers mostly dealt with controversial diet procedures such as alkaline fasting, detox diets, and low-carb diets. Neither the influence of acid-forming foods on the acid–base balance, nor the existence of so called “slags” and consequent detox diets are evidence-based concepts and methods [32, 33]. Strong carbohydrate restrictions should also be viewed critically. Seidelmann et al. could show in their study that both a low carbohydrate intake (< 40% of energy) and a high carbohydrate intake (> 70% of energy) are associated with an increased risk of mortality [34]. None of the diet methods mentioned are recommended by the German Nutrition Society for long-term weight loss. Long-term use of non-evidence-based dieting methods can be associated with health risks, as vital nutrients could be supplied in insufficient quantities. It should also be addressed that very strict diets with rigid eating behavior and low protein and calorie intake can increase the risk of eating disorders.

In addition to several findings, our analysis also had limitations. Interpretations were based on course descriptions which were published on the websites of adult education centers. We are aware that the course descriptions may not reflect all characteristics and details of the content conveyed. Within our analysis period there was still no description for some courses; for other health courses, descriptions were partly no longer available.

Geographically, the search radius of our analysis was limited to 80 km based on the ten largest German cities by population. Rural, less densely populated regions outside this search area could not be included. Therefore, courses with regard to CAM and nutrition could potentially differ.

Detailed information on the course instructors and their professional skills were also not regularly found. In a quarter of the cases, despite naming the course instructor, no information about his professional qualifications was given. In addition, the diversity of offers on the market for training and further education without any statutes on binding content made the comparison even more difficult.

## Conclusion

In summary, the courses of German adult education centers concerning CAM and nutrition/diets show a strong preponderance of alternative topics, which are incompatible with evidence-based medicine or whose effectiveness has not been proven or refuted; some of them are even associated with health risks. Moreover, many instructors belong to professions which are not trained in scientific evaluation of methods.

In fact, many courses contribute to the popularity of alternative and non-evidence-based notions in the public and do not fulfill their function in adult education.

In contrast, to improve health literacy in the population, courses should focus on evidence-based health topics. Courses on CAM might even encourage people to develop critical thinking about medical topics. For this purpose, a differentiated presentation of the methods with an explanation of evidence, benefits, but also risks and side effects as well as a comparison with other methods, would be necessary. From our analysis, we did not find hints on such a differentiated approach.

The selection of course instructors should be critically reviewed. Most of the courses are conducted by NMPs. Studies have already proven deficiencies in the medical training provided by NMP schools [9]. In fact, NMPs in key positions in health education must be viewed carefully. Physicians, psychotherapists, and physiotherapists as well as certified nutrition experts are rare among the course instructors. To improve the quality of course content, these groups should be preferred as instructors.

Considering the relevance of health literacy defined as the ability to find, understand, and use health information to make health-related decisions in modern society the importance of health education is obvious. Adult education courses have the important task of establishing health literacy for a broad part of the population.
